# Gendering of Indian judiciary as a roadmap towards an equitable legal system and progressive gender-sensitive jurisprudence

**DOI:** 10.3389/fsoc.2025.1475043

**Published:** 2025-03-26

**Authors:** Harleen Kaur

**Affiliations:** Campus Law Centre, Faculty of Law, University of Delhi, New Delhi, India

**Keywords:** Indian constitution, female judges, gender inequality, gender-variant persons, justice system

## Abstract

Given that women constitute 50% of the global population, it is imperative that all professions that influence social progress, including the judiciary, acknowledge and include this demographic. Their inclusion in all professions is not only essential for paving the path to a just and gender-equal society but is also imperative for the true social progress of a Nation. This holds good for the Judiciary as well where in the inclusion of women judges not only brings in diverse perspectives leading to more legitimate and comprehensive judgments, but also reflects broader societal changes toward creating a more equitable legal system. It is noteworthy that Indian courts have continually progressed toward ensuring the involvement of women in the administration of justice, and indeed, the engagement of women judges within India’s criminal justice system in recent years has played a crucial role in facilitating the necessary societal changes through their landmark decisions consistently advancing constitutional values and norms. However, a significant gap still remains which requires more affirmative actions to achieve a balanced and an inclusive judiciary that upholds the rule of law and the principles of equality enshrined in the Constitution of India. Consequently, this research paper, while providing an insight into the current gender ratio in India’s judicial system, though primarily highlights the importance of having enough women on the bench, both as a constitutional requirement and as a critical step toward creating a trustworthy justice system however, it also emphasis on the need to appoint gender-variant persons in the Judicial institutions laying down the foundation for a truly inclusive and equitable society.

## 1 Introduction

### 1.1 Need for gendering judiciary

Gender Justice has garnered recent attention in law and justice, particularly in the developing world, where discrimination and inequality based on gender are a common phenomenon that needs to be addressed in line with the universally accepted Gender equality goal outlined in the ‘Charter of the United Nations’ which unequivocally emphasizes that gender equality, women’s rights, and women empowerment are essential for creating a fairer and a more just world for everyone and the same gets strengthened by a number of commitments taken which includes, among others, ‘The Convention on the Elimination of all Forms of Discrimination against Women (CEDAW)’, ‘the Beijing Declaration and Platform for Action’, various resolutions and decisions of the ‘United Nations General Assembly’, ‘the Security Council, ‘the Economic and Social Council’, as well as the ‘Commission on the Status of Women’. Further, the ‘Sustainable Development Goal 5 (SDG 5)’ of the 2030 Agenda, while reaffirming that gender equality is a condition sine qua non for sustainable development and at the same time, an indispensable outcome of it, calls upon its Member States to mainstream gender into their legislation and policies and to adopt special measures to promote gender equality and empower women and girls. When considering India, the trajectory toward achieving gender equality in various aspects of civil society, including the judiciary, underwent a considerable transformation with the appointment of ‘Anna Chandi’ as the first female judge of a High court in 1937 and subsequently with ‘Fathima Beevi’ becoming the first female judge of the Indian Apex Court ie, the Supreme Court (SC). Consequently, women gained entry to the Supreme Court Bench, which served as a platform for them to make a lasting impact. This development effectively dismantled gender stereotypes and structural obstacles that had previously impeded the advancement of women within the legal community in India. Justice ‘Sujata Vasant Manohar’ assumed the position of Supreme Court Judge 5 years after the tenure of Justice ‘M. Fathima Beevi’, a notable female justice.

With 276 judges appointed since attaining independence, the number of women judges in Supreme Court (SC) has been relatively low at 11, i.e., an abysmal 4% of the total 276 judges (Gauri, 2024). In fact, a perusal of the official website of the Supreme Court as on 30th November, 2024 reveals that out of a total of thirty Four (34) justices (1 Chief Justice and thirty three (33) judges) at present, number of women at the SC has reduced to 2 from 3 following the retirement of a woman Justice in early September, 2024. Further, as per the information available on the official websites of 25 High Courts (HC’s) across various Indian States and Union Territories, women Justices make up 13.76% of all sitting judges in all high courts, i.e., 96 out of 719 judges, as shown in Table 1.

**Table 1:** Information about number of sitting women Judges in various HC’s as accessed on November 30, 2024.

Serial number	High court	Number of sitting women judges
1	Allahabad	3
2	Andhra Pradesh	5
3	Bombay	11
4	Calcutta	6
5	Chhattisgarh	1
6	Delhi	9
7	Guwahati	4
8	Gujarat	7 judges+ I chief justice
9	Himachal Pradesh	1
10	Jammu, Kashmir, Ladakh	2
11	Jharkhand	1
12	Karnataka	8
13	Kerala	4
14	Madhya Pradesh	2
15	Madras	12
16	Manipur	1
17	Meghalaya	0
18	Orissa	1
19	Patna	1
20	Punjab & Haryana	13
21	Rajasthan	3
22	Sikkim	1
23	Telangana	8
24	Tripura	0
25	Uttarakhand	0

Infact, a perusal of official websites of Various HC’s as on 30th November, 2024 by the researcher revealed almost a similar status as mentioned in the SC’s ‘State of the Judiciary Report 2023’, i.e., 13.4% proportion of female judges, in various HC’s and 9% in SC with no female justices in the High Courts of Uttarakhand, Patna, Tripura, Meghalaya, and Manipur (Supreme Court, Centre for Research and Planning, 2023). The only visible changes were a minimal increase in representation of female judges from zero (0) to one (1) in the Patna HC and from 10 to 11 in the HC of Bombay, in addition to the appointment of a female Chief Justice in the HC of Gujarat. However, with Punjab and Haryana HC having the highest number of female judges, which is 13 at present, and the lowest being zero (0) in the High Courts of Uttarakhand, Meghalaya, and Tripura, super sincere efforts would be required to close the gap. This wide disparity reflects upon the lack of significant efforts to increase women’s representation in Courts in the last seven decades. With no female Chief Justice of India (CJI) and just one women chief justice of Gujrat High court, the gender gap in India’s highest court is very wide. In 2027, however, there is a possibility that India may see its first woman Indian Chief Justice, i.e., Justice ‘Nagarathna’ although her tenure would only last for 36 days. In so far as the representation of women judges in subordinate courts is concerned, as per the data revealed by SC’s ‘State of the Judiciary ‘Report 2023 (Supreme Court, Centre for Research and Planning, 2023),’ women constitute 36.3% of subordinate Court judges with Goa having the highest proportion of female judges, i.e., 70% at the district court level, followed by Meghalaya (62.7%), Sikkim and Telangana also come close to with 52.4% & 52.8%, respectively in addition to 13% proportion of female judges, in various High Courts and 9% in Supreme Court with no female justices in the High Courts of Uttarakhand, Patna, Tripura, Meghalaya, and Manipur.

Further, according to the World Economic Forum (2022), India is ranked 48th out of 146 countries in the Political Empowerment dimension, which measures the percentage of women in parliament and in ministerial positions. Currently, the proportion of female members in the Indian Parliament is at approximately 14.4%, which is the highest recorded thus far. In fact, with the aim to advance gender equality and women’s empowerment in a patriarchal Indian Society, the Indian Parliament has very recently cleared the ‘*Nari Shakti Vandan Adhiniyam’* ie,‘Women’s Reservation Bill’, 2023 which mandates that 33% of Lok Sabha seats (Lower House of the Parliament), the ‘Vidhan Sabha’ seats (Provincial Legislative Assemblies), and the Delhi assembly seats be specially reserved for women. Nevertheless, the execution of the allocation of the seats has been postponed until the completion of the Census and a delimitation procedure (The Constitution (106th Amendment) Act, 2023). The passage of this measure has also ignited a discussion suggesting the implementation of a 33% quota for women in the judiciary to address the existing gender disparity in our judiciary and to serve as a catalyst for other necessary reforms. However, at the same time, several concerns have also been raised regarding its feasibility and impact ranging from undermining meritocracy, resulting in prioritizing gender over qualifications, leading to less qualified individuals filling positions apart from these quotas being influenced by political considerations, rather than judicial merit. However, these concerns can be taken care of by reforms in legal education and professional development, inclusion of independent judicial committees, with representation from legal professionals, academia, and non-partisan entities, to ensure merit-based selection of candidates, in addition to regularly reviewing of the impact of quotas to assess if they are leading to the desired outcomes, in addition to introduction of system of feedback and mentorship programs to ensure that women not only occupy positions of power but also excel in them. It cannot be denied that by doing so, India’s status will be elevated, placing it among the progressive nations in terms of gender parity and which would also be instrumental in breaking the glass ceiling that many women face, particularly in fields where they have been historically excluded or marginalized. In India, not only do women make up only a small proportion of the judiciary, but similar gender imbalances are found in politics and other leadership roles too. However, at the same time, the above proposal for a 33% quota for women and gender-variant persons in the Indian judiciary has been a topic of significant debate, too.

## 2 Method and material

This research is purely doctrinal in nature and is based on primary and secondary sources and explores the interplay of various feminist theories which call for the restructuring of legal practices and the inclusion of diverse perspectives for the purpose of analyzing how different feminist perspectives interact with challenges and shape legal principles and how adoption of these theories in the alternative and in combination, provide for a roadmap for creating a more inclusive, equitable, and a just society. Further, the author by reviewing the existing literature and exploring the path-breaking decisions given by the Constitutional Courts of India, illustrates the significant role that female judges can play in the Indian Judiciary toward developing progressive and gender-sensitive jurisprudence. Though the primary focus of the research paper is on advancing the representation of females in judicial institutions in India, however, the vacuum that can be noticed in the appointment of gender-variant persons in judicial institutions has also been highlighted in this research. With respect to statistics pertaining to the representation of women as judges in various HCs and the SC, the same has been collected and verified from the official websites of such Courts along with a perusal of Supreme Court’s ‘State of the Judiciary’ Report 2023′ released by the SC of India (Supreme Court, Centre for Research and Planning, 2023).

## 3 Results

The comprehensive analysis of the research reveals that the establishment of a more gender-diverse bench in Indian Courts will not only lead to a more inclusive judicial system but will also help in ensuring a robust legal system that aims at promoting true gender justice and equality at all levels of society. Undoubtedly, Women’s representation in the Indian judiciary has come a long way, but it remains far from being equal to that of men. Similarly, various legislative and judicial reforms in India set the tone for the Inclusiveness of the LGBT+ community and are worth celebrating. However, despite all these measures, the LGBT+ community is battling hard in their quest for social upliftment. Thus, their inclusion in the process of imparting justice might just bring a shift in the progressive thinking which is much needed to accord true sense of equality to these persons. Consequently, the research puts forward various suggestions and recommendations for bringing gender diversity to the judiciary in order to achieve an inclusive and equitable society in the light of the emergence of various feminist theories which not only pave the way for a responsive legal system dismantling of traditional gender biases in legal institutions but also provides for frameworks to address the unique challenges and barriers faced by women, gender-diverse individuals at the intersections of multiple identities fostering a more inclusive, just, and equitable judicial environment for gender-inclusive representation.

## 4 Discussion

### 4.1 Feminist theories: comparison, synergy and their response to gender justice and equality in legal and judicial frameworks

As legal systems around the world continue to evolve, the evolution and integration of various feminist theories such as ‘*feminist legal theory’, ‘difference theory’, ‘queer theory’, ‘equality theory’ and the theory of ‘intersectionality’* not only laid down the foundation for a more inclusive and equitable socio-legal landscape by providing insights into how gender dynamics can shape legal systems but also offered important pathways for transforming the Judiciary into a space for promoting gender equality, respecting diverse gender identities, and thus laid a challenge to the system entrenched with patriarchy. While these theories focus on gender in different ways yet, in application they appear to be complementary and often operate together in the global fight for inclusive policies and inclusive judicial systems addressing the specific needs and challenges essential for the elimination of multiple layers of discrimination. For instance, demonstrating that no one’s experience of oppression or privilege is solely defined by one’s identity like gender alone, *the theory of intersectional feminism*, a foundational work of kimberlé crenshaw, examines how various forms of oppression (e.g., gender, race, class, sexuality) intersect to create complex systems of inequality and thus the theory advocates for a nuanced, context-specific approach to activism and policies to address systemic inequalities affecting marginalized groups and their access to justice (Crenshaw, 1989). Similarly, resting on the argument that women and other marginalized groups (e.g., LGBTQ+ individuals) have distinct experiences due to their unique social roles such as oppression and cultural realities, the *difference theory* propagates gender differences to be recognized and valued in legal systems, whereas the *equality theory* in contrast, advocates for equal treatment and rights for all individuals, regardless of gender, race, or other social identities and emphasizes gender-neutral legal frameworks, ensuring that everyone has equal access to justice. Thus, it can be seen that though the difference theory and equality theory in feminist jurisprudence offer distinct yet complementary perspectives on how gender diversity impacts legal systems, each contributes to the broader discourse on gender justice and equality within the judicial framework. Likewise, a perusal of feminist legal theory and queer theory reveals how combined efforts of both these theories advance gender diversity aimed at challenging entrenched socio-legal norms and power dynamics, in order to promote a society where all gender identities and expressions are recognized and respected. The *feminist legal theory* emerged as a response to the historical exclusion of women and gender minorities from legal systems and how laws perpetuate gender inequality and highlights the reforms required to challenge the patriarchal legal systems. *Queer theory*, while often linked with feminist legal theory, deconstructs the heterosexual and cisgender norms embedded in legal structures and advocates for inclusive laws that recognize the spectrum of gender identities and sexual orientations. ‘Judith butler, Michel Foucault, and Eve Kosofsky Sedgwick’ are key contributors to the development of queer theory. It will be apposite to mention here that these theories, when applied in combination, contribute toward a more equitable and inclusive world for people of all genders.

It will also be pertinent to mention that the application of the above theories within the judicial frameworks of various legal systems has indeed offered a progressive approach to achieve gender justice and equality by making these institutions not only more inclusive for diverse gender identities and expressions but also responsive to the unique experiences and intersectional realities faced by such marginalized groups. Therefore, at this juncture, perusal of some landmark judgments and developments, both at the National and International level becomes essential as adoption of these theories, in alternative and in combination as well really helped in achieving inclusive and equitable standards. For instance, the U.S. Supreme Court (SC) ruling on same-sex marriage in ‘*Obergefell v. Hodges’ (2015),* wherein it was held same-sex couples should have the same rights as heterosexual couples, when viewed from the theoretical lens of scholars, seems to have drawn from ‘queer theory and feminist legal theory’, emphasizing the legal recognition of diverse family structures and gender equality. Similarly in the case of ‘*Whole Woman’s Health v. Hellerstedt’ (2016),* where in the U.S. SC struck down restrictive abortion laws in Texas that disproportionately affected women’s access to reproductive health again is a decision reflecting a broader understanding of women’s experiences with reproductive health and access, in alignment with‘d*ifference theory’*, calls for laws to recognize these gendered realities. In South Africa also, in the cas*e* of *‘Minister of Home Affairs v. Fourie’ (2005),* the South African Constitutional Court emphasizing the need for legal recognition of diverse sexual identities and partnerships within the legal system in alignment with *feminist legal theory* ruled in favor of same-sex marriage thereby by challenging traditional patriarchal norms that historically defined marriage. Further in the case of ‘*Mankayi v. Anglo Gold Ashanti Ltd.’ (2011),* the South African Constitutional Court addressed the right of the miners suffering from occupational diseases and their aability to claim compensation considering the intersectionality of race, class, and economic status within the context of systemic labor and health injustices. Similarly, the Canadian Supreme Court aligning with the core principles of intersectionality in the case of ‘*Vriend v. Alberta’ (1998)*, ordered for that sexual orientation be read into the legislation as a protected category and thus advocated for more nuanced and inclusive approach recognizing structural inequalities perpetuated by such legal omissions.

Upon examining Indian jurisprudence, which addresses intricate kinds of discrimination and advances substantive equality, there is a clear evidence of progressive application of feminist theories and intersectional analysis. For instance, in the case of – *‘Sarla Mudgal v. Union of India’ (1995)*, wherein the issue of bigamy was addressed in the context of Hindu marriages by addressing the intersection of personal laws and women’s rights, sparked national discourse on the Uniform Civil Code, in the complex framework of India’s pluralistic legal system. Similarly, another case, i.e., *‘Vishaka v. State of Rajasthan’ (1997),* aligning with the key aspects of radical feminist theory, which views sexual violence as a tool to maintain patriarchal control over women, resulted into framing of comprehensive guidelines to address sexual harassment at workplaces taking into account women’s experiences of gender-based violence. Another demonstration of complex interplay of difference theory (recognizing the exclusion of women from religious practices) and equality theory (ensuring women’s equal access to religious spaces) was observed in the *‘Indian Young Lawyers’ Association v. The State of Kerala’* (2019) when the Court acknowledged that gender exclusion was a systemic problem ingrained in religious practices rather than only being an individual one. Further, drawing on intersectional understandings of sexuality and gender and reflecting queer theoretical perspectives, the Supreme court in case of ‘*Navtej Singh Johar v. Union of India (2018)’,* decriminalized the same-sex relations and upheld the that LGBTQ+ rights. Once more, in the matter of ‘*Suchita Srivastava v. Chandigarh Administration (2009)*’, in which a mentally challenged woman had been raped, the court upheld the survivor’s right to bodily autonomy while acknowledging her vulnerability and affirming the significance of agency, in accordance with the tenets of difference theory.

In another significant ruling, ‘*Laxmi v. Union of India’ (2015)*, the SC recognized the intersectional vulnerabilities of acid attack survivors and issued stricter regulations on the sale of acid thereby addressing patriarchal violence and unique trauma experienced by such women. Further the SC in the case of ‘*Independent Thought v. Union of India’ (2017)* by criminalizing marital rape of minor, too marked a progressive shift in Indian legal landscape reflecting key feminist principles advocating for bodily autonomy and substantive equality.

Although various judgments reflecting these theories propagate for equal representation of gender diverse people in all sects of life, the appointment of gender-diverse judges globally has had a transformative impact on the judicial system and their contribution to the broader discourse on gender justice and equality has been a great win for various judicial systems across the globe. For instance the appointment of ‘Justice Sonia Sotomayor’, as the first Latina Supreme Court Justice in the year 2009 as well as of ‘Justice Ketanji Brown Jackson’s’, the First Black woman to hold the position in U.S. SC in 2022 broke long-standing historical conventions. Further with gender-diverse appointments, such as of ‘Justice Rosalie Abella’ in Canada’s Supreme Court and ‘Victoria Kolakowski’, in the United States’ made historic accomplishments in promoting the inclusion of LGBTQ+ rights in the legal system and addressing gender and racial human rights issues. Similarly, the appointment of ‘Ming-Ko Hsiao’ to the Taipei District Court in Taiwan in 2017 and the appointment of Judge ‘Phyllis Frye’ in the United States in 2010, contributed to increased societal acceptance of transgender people and marked a turning point in the legal recognition of transgender rights in these nations.

Talking about India, the appointment of female judges such as ‘Justice M. Fathima Beevi,’ ‘and Justice Indu Malhotra,’ was also a significant milestone toward a more inclusive and representative judiciary in view of under-representation of women in India’s judiciary. Further appointment of ‘Joyita Mondal’ as India’s first transgender judge in the West Bengal Judicial Service in 2017 not only broke down all conservative barriers but also demonstrated the possibility of social and professional inclusion for transgender individuals in a country where transgender people often face marginalization. Indian judiciary, however, still remains largely cisgender and male-dominated with a complete absence of individuals from non-binary and transgender communities in the Apex court or various High Courts in India despite progressive judgments like ‘*NALSA v. Union of India’ (2014) and ‘Navtej Singh Johar v. Union of India’ (2018)* wherein the SC by recognizing the fundamental rights of transgender and LGBTQ+ individuals not only marked historic progress for transgender and LGBTQ+ rights in India but also laid the foundation for legal gender identity recognition, equality, and dignity under the Indian Constitution. Similarly, disproportionately low representation of women even from privileged, upper-caste backgrounds, with a complete absence of Dalit, Adivasi, and disabled women is not just a matter of numbers but a deeper issue of systemic exclusion when analyzed from the lens of intersectional analysis exposing not only the gaps between progressive legal principles and systemic judicial biases but also lack on the part of Indian judiciary to ensure structural reforms for genuine inclusivity and thus calling for urgent reforms in the form of adoption of affirmative action’s such as reservations, transparent recruitments, and active sensitization to reflect not only the diversity of the society it serves but to deliver true justice that requires that marginalized voices are heard and represented not just in courtrooms but on the bench itself.

### 4.2 Female judges in India and their prominent judgments

Each judgment type, whether dissenting, distinguishing, or concurring, has the potential to influence Indian jurisprudence by its diverse legal opinions, additional or alternative interpretations, and subsequent application of the law. The Judicial Consensus not only establishes the Court as an institution but also provides unquestionable clarity and predictability. This elucidates the reason behind the frequent occurrence of unanimous rulings by both the HC and the SC of India. Yet the dissent perspectives, which are frequently eclipsed by the thoughts of the majority, are beneficial to the accountability of judgments, the evolution of the law, and the reform of the law. Dissent not only showcases the existence of multiple viewpoints but also serves as a jubilant event for the democratic ethos, which thrives in a setting that fosters and deliberates on conflicts.

#### 4.2.1 Landmark dissent verdicts

It is noteworthy that a female judge may have a higher tendency to dissent when she is the sole woman on the bench, as her non-differing opinions are less likely to be included in the majority decision. However, as the number of women on the panel increases, the likelihood of female judges dissenting decreases. Hence, it is imperative that there is a greater emphasis given to inclusivity of female judges within the judiciary. A greater number of female judges will facilitate the inclusion of claimants’ arguments in gender-coded matters inside courts, thus contributing to the establishment of robust, autonomous, inclusive, and gender-sensitive institutions. It cannot be denied that the majority rulings play a great role in shaping law nevertheless the impact of dissenting judgments can also never be overlooked [‘*A.K Gopalam v. State of Madras*’(1950); ‘*Kharak Singh v. State of UP’* (1964); ‘*ADM Jabalpur v. Shivkant Shukla*’ (1976)]. In fact, a perusal of the history of Indian Supreme Court judgments reveal that dissent is being recognized as a *“symbol of a vibrant democracy,”* often containing a better solution by default and thus, later becomes the law of land. Therefore, it becomes imperative at this stage to look into some of impactful dissenting opinions given by women judges in various courts in India. In the ruling of ‘*Kaushal Kishor v. State of Uttar Pradesh & Ors’* (2023) which involved issue pertaining to hate speech, Justice Nagarathna expressing her views on the matter in her dissenting verdict stated that in a democracy that values human dignity, the freedom to speak and express oneself should be used in a way that safeguards and advances the rights of fellow citizens. Regardless of its substance, hate speech deprives individuals of their inherent right to dignity (‘*Kaushal Kishor’*, Para 16.3). While citing the case of ‘*Amish Devgan v. Union of India’* (2021), wherein the court declared that ‘hate speech’ is contradictory to and inconsistent with the fundamental principles of human dignity, she additionally asserted that hate speech occurs when an individual’s misuse of the freedom of expression infringes upon the basic rights of another person. (Paragraph 25). The Preamble of our Constitution encompasses the fundamental principles of equality, liberty, and brotherhood. ‘Hate speech’, as previously mentioned, undermines all of these fundamental ideals by identifying a society as being unequal. It also infringes upon the brotherhood of citizens from all backgrounds, which is essential for a unified society built on diversity and multiculturalism, as seen in India, also known as Bharat.

Similarly, while reviewing the government’s demonetization decision in November 2016 in the case ‘*Vivek Narayan Sharma v. Union of India*’ (2023) Justice ‘B V Nagarathna’, the junior member of a five-judge Constitution Bench, determined that the Reserve Bank of India (RBI) showed a deficiency in impartial reasoning. The fact that the RBI did not independently exercise its jurisdiction is visible from the Central Government’s use of language such as “*as desired*” and “*the government had recommended*” to withdraw the legal tender of existing Rs 500/− and Rs 1,000/− notes along with the mention of “*recommendation has been obtained*.” It is also a matter of fact that the RBI had insufficient time to devote its thoughts to such a grave matter. This observation was made in consideration of the 24-h duration of the demonetization process pertaining to the entire series of Rs. 500 and Rs. 1,000 bank denominations. It will be worthwhile mentioning here, that J. Nagarathna’s critique of the RBI’s perceived subordination to the Government not only brought into forefront, the important concerns about the independence of regulatory bodies and the executive overreach, but also the importance of procedural integrity in maintaining public trust. Through her dissenting opinion not only she advocated for a stronger parliamentary oversight on critical economic decisions and also stressed upon the need to ensure transparency and accountability in such decisions adhering to Constitutional principles.

In another well-known case, i.e., ‘*Indian Young Lawyers’ Association v. The State of Kerala’* (2019), it was opined that the restriction on access to women inside the Sabarimala temple during their menstrual cycle is a violation of the constitution. Consequently, the court revoked the prohibition imposed on women between the age group of 10 and 50 years from attending the temple. In contrast to the majority ruling in the Sabarimala case, Justice ‘Indu Malhotra’, the sole female judge who expressed opposition, upheld the well-established principle that state courts should abstain from making judgments regarding the prohibition of religious rituals unless they pertain to societal vices like Sati. Furthermore, she emphasized the importance of limiting the influence of courts in matters pertaining to religious beliefs. This was crucial to prevent a surge of appeals that might have potentially damaged India’s secular structure and its diverse population. Justice Indu Malhotra’s dissenting opinion was widely commended by the legal profession for its adherence to legal principles and for her display of bravery in challenging prevailing public sentiment. Though her dissent did not prevail, but it did bring to light, the intersection of religion and gender rights in India and sparked a discourse about the delicate balance between respecting religious beliefs and ensuring they align with modern constitutional values. This case became a catalyst for examining other religious customs through the lens of gender equality prompting a broader discourse on women’s rights in religious spaces and equality in places of worship.

Further, ‘Justice R Banumathi’, while hearing for ‘*Mukesh & Anr v. NCT of Delhi & Ors’* (2017) upheld the death sentence imposed on the defendants involving the “Nirbhaya” gang rape case subsequent to their appeals seeking reconsideration of their respective death sentences. In her distinct yet concurring opinion, ‘Justice Banumathi’ not only engaged in meticulous deliberation over the severity of the offense warranting capital punishment but also contemplated the broader societal framework within which the Court rendered its decision. The justice emphasized that the crime serves as a demonstration of the necessity for broader societal transformation in order to attain gender justice. The prevailing belief that the testimony of a victim must be substantiated by additional evidence should be eliminated. Drawing a parallel between a rape victim and an accomplice is an affront to the concept of gender. If the victims are vulnerable women, children, or elderly individuals and the accused exhibits a corrupt mindset, committing a crime in a malevolent manner, it is appropriate to express no remorse for the accused and be sentenced to death (Mittal and Jain, 2023).

It is imperative to note that such observations from judges like ‘Justice Bhanumathi’, not only contribute to policy debates and need for a cultural shift to address deep-rooted misogyny and violence, but also paves the way for a more responsible Judiciary in fostering social responsibility.

#### 4.2.2 Landmark majority verdicts

There have been various significant rulings with women judges on the bench that demonstrates the vital role played by Indian women judges in shaping the trajectory of justice. In cases involving sexual abuse, coerced marriage, abortion, and rape, for instance, Indian women judges have played a crucial role in determining the course of justice and have had a significant impact on the criminal justice system. They have also had a significant impact on the ongoing evolution of family law, allowing for the establishment of more progressive interpretations and acknowledging the significance of individual autonomy and freedom within the context of marriage. Some of these judgments can be seen as under:

The Division Bench of the Supreme Court, which included ‘Justices Ruma Pal, Arijit Passayat, and CK Thakkar’ in the case of ‘*A. Jayachandera v. Aneel Kaure’* (2005), ruled that mental cruelty must be considered in relation to the marital ties common in the community to which the individuals belong with their social standing, living conditions, and values. Cruelty is an act where one spouse’s actions cause serious physical and mental trauma to another spouse. It is crucial to look into abusive traits and the effects they cause. It is worth mention that by expanding the definition of cruelty and emphasizing that it is not just about physical harm but includes mental and emotional harm, this judgment reminds society, the value of emotional well-being in marriages and the need to encourage respectful relationships thereby paving the way toward a healthier future for individuals and families.

In the case of ‘*Vinitha Saxena v. Pankaj Pandit’*, (2006), a bench of the Apex Court comprising of ‘J. Ruma Pal and J. A.R. Laxmannan’, ruled in favor of the wife by granting a divorce. Based on the given facts in the court, the accused spouse was diagnosed with Paranoid Schizophrenia, a psychiatric disorder that was unknown to both the wife and her parents before the marriage. The court determined that the determination of mental cruelty is not contingent upon the frequency of such occurrences or the magnitude of abuses but rather on its detrimental effect on the psychological disposition necessary for fostering a harmonious domestic environment. If the insults, complaints, and reproaches were of a less serious nature, the court may need to determine if the victim’s spouse has endured them for a sufficient duration to cause such intense harm and suffering that the accused spouse reasonably believes it is impossible to maintain the marital home.

Furthermore, although there is no formal recognition of a second wife under Indian Legal System, the legal interpretation of established legislation regarding the entitlement of the second wife to maintenance was courageously addressed in the ‘*Pyla Mutyalamma v. Pyla Suri Demudu’ c*ase@ *Satyavathi,* (2011). This judgment addressed the issue of whether women who have been found guilty of bigamy under section 125 of the Code of Criminal Procedure (CrPC), 1973, are not entitled to maintenance. In reaching this conclusion, the division bench of Justices ‘Gyan Sudha Mishra and Harjit Singh Bedi’ determined that section 125 of the Cr. PC operates under the supposition of a de facto marriage rather than a de jure marriage. As a result, the spouse who asserts that his pre-existing marriage renders the second marriage null and void must provide the court with sufficient evidence to support his claim. In the event that the spouse is unable to provide such documentation, the court will presume that the marriage is valid and will have the authority to award maintenance.

It can be seen, the above mentioned judgments serve as a crucial reminder of the importance of honesty, commitment, and respect essential to build a foundation of trust and understanding in marital relationships ensuring that those who are wronged receive the protection and support they deserve.

In the case of ‘*State of Madhya Pradesh v. Jogendra & Anr’* (2022), a three-judge SC bench which included Justice ‘Hima Kohli’ stated that the High Court made a mistake by ruling that the demand for money for constructing a house cannot be considered as a dowry demand. The judges pointed out that the Dowry Act defines ‘dowry’ broadly to include any kind of property or valuable security. Therefore, by including monetary demands made by the husband or in-laws, such as for constructing a house, within the meaning of Dowry, such decisions reinforce the need to view dowry as a continuing social evil, urging collective action against both overt and covert forms of this practice.

Similalrly, in ‘*Smt. Pratibha Singh v. Mr. Vineet Kumar’* (2023), the Delhi High Court, speaking through Justice ‘Pratibha M Singh’, while deciding a petition pertaining to a matrimonial dispute involving a woman, underscored the importance of maintaining a balance between the rights of both parties within a family unit. Consequently, the court determined that a daughter-in-law’s right in the household is not absolute and should not be exclusive to the in-laws. Thus, this ruling contributes to a broader societal understanding of marital responsibilities, financial accountability, and the need for equitable treatment in family law cases.

Further, in *‘Smt. Chetna Rathee v. Sh. Chahit Kundu’* (2023), a case decided by a single judge bench of Justice ‘Rekha Palli’, established that when the Court is addressing petitions related to Family Law and the parties involved are already in conflict, it is predicted that the Court should not adopt excessively technical conduct and restrict the parties’ right to be cross-examined in a hasty manner, even if the dispute is to be decided promptly.

In the ‘*Joseph Shine v. Union of India’* (2018) case, a five-judge Bench of the SC including Justice ‘Indu Malhotra’, penned an undivided judgment whereby Section 497 of the Indian Penal Code (IPC) 1860, was invalidated. This ruling effectively decriminalized adultery, deeming it unconstitutional, outdated, and clearly arbitrary. Justice ‘Indu Malhotra’ in particular observed that adultery should be tried as a civil wrong, and can only be a ground for securing divorce. Therefore, the court in the instant case upheld the constitutional morality of the time and acknowledged the significant impact of this doctrine within Indian judicial system. Again here it can be seen that by promoting the idea that women are autonomous individuals capable of making their own choices and should not be subjected to discriminatory laws, this judgment recognizes women’s autonomy in marital relationships thereby challenging deep-rooted patriarchal norms and paves the way for more progressive interpretations of gender roles and marital freedom in India.

Also, it is pertinent to note, that in last few years, the Courts in India have been recognizing the right to reproductive autonomy as one flowing from the right to take decisions for one own self thereby bringing the same at par with the right to privacy and dignity under Articles 14 and 21 of the Indian Constitution. In the landmark case of ‘*Pratibha Gaur v. NCT of Delhi’* (2021), the Delhi HC, speaking through Justice ‘Jyoti Singh’ granted the permission to the plaintiff to undergo the surgery after 28 weeks of pregnancy stating that reproductive choice free of discrimination, coercion and violence is an essential component of reproductive rights and continuation of a pregnancy knowing the baby has a high risk of major defects and difficulties and if born be largely dependent on the clinical condition and quality of medical care would have a “deleterious impact on the petitioner’s mental wellbeing.” Therefore, responsible for making the ultimate decision regarding whether or not to undergo surgical abortion rests with the petitioner which she would do at her own risk and according to her own duty.

Likewise, in another instance before the Delhi HC ‘*Mrs. X v. GNCTD & Anr’* (2022), a single bench of Justice ‘M Pratibha Singh’ also ruled that in pregnancy cases involving fetal abnormalities, the mother has the final say. The bench also emphasized that medical boards are obligated to provide qualitative reports in such situations. Moreover, it was noted that in instances involving the termination of pregnancy, the perspective of the medical board holds significant importance in aiding the court. Consequently, it is imperative that such an opinion is comprehensive in form and devoid of any ambiguity or fragmentation.

Thus, in addition to advocating for women’s rights and health as a crucial component of social justice reforms, this ruling lays the groundwork for a more equitable and humane healthcare system destroying the legal vestiges of patriarchy.

Similarly, the journey of Indian Courts with female judges playing a significant role on the bench, starting from recognition of sexual orientation as an intrinsic component of self-identity rather than an anomaly and playing a crucial role in protecting marginalized sections from various forms of violence is noteworthy too and which can be manifested from a bare reading of the following notable rulings: -.

In ‘*Navtej Singh v. Union of India*’ (2018), The SC’s constitution bench, which included Justice ‘Indu Malhotra’, unanimously read down Section 377 of the IPC, which penalized same-sex relationships by holding that it violative of various fundamental rights of a citizen including the ‘right to privacy, equality, freedom of expression, human dignity, and protection from discrimination’, thereby decriminalizing consensual same-sex relations between consenting adults. It was further held that the inclusion of sexual orientation as an intrinsic component of self-identity is an undeniable reality, notwithstanding its limited impact on a small portion of the population. J. Malhotra also asserted that homosexuality should be regarded as a manifestation of sexuality rather than an anomaly and the entitlement to privacy encompasses not simply the entitlement to solitude but also encompasses “*spatial and decisional privacy.”* Further, she closed her viewpoint by asserting that it is imperative for history to extend an apology to individuals belonging to the LGBT community and their families, acknowledging the prolonged period of time it took to address the injustices and marginalization they have endured throughout history.

Similarly, in the case of ‘*Neetu Singh v. Telegram* (2022)’, where in the Plaintiff’s copyrighted material such as books and video lectures were being illegally circulated on various Telegram group, Justice ‘Pratibha M. Singh’ assuming the role of single bench granted an interim injunction and ordered Telegram to provide details regarding the channels and devices employed for the distribution of infringing content. This judgment assumes importance in view of the ever growing and ever changing technology, especially digital technology wherein protection of intellectual property rights of an individual are under a constant threat and which need to be protected at all costs.

In ‘*Rattan Chand v. Askar Nawaz Jung Hira Chand* (1991)’ case, Justice ‘Fathima Beevi’ underscored the significance of public policy in determining the legitimacy of a contract, asserting that any agreement lacking lawful purpose or consideration is rendered null and void. According to her, if judges deem something to be in violation of public policy, the agreement becomes null and void. Thus, judgments like these, entrust Indian courts with the responsibility to ensure contracts align with public policy. Contracts with unjust or immoral considerations are deemed void thereby strengthening the ethical foundation of Indian contract law.

In ‘*People’s Union for Civil Liberties v. Union of India’* (2003), Justice ‘Ranjana P. Desai’, a member of the Three Judge Bench, ruled that the voter must have the option to select option of None of the Above (NOTA) in a functioning democracy and thus directed the Election Commission to establish the option of NOTA in the electronic voting machines (EVMs). It was also observed that voter being provided with an option of NOTA will also compel the nomination of a viable candidate by the political parties. This ruling significantly impacted the Indian democracy by reinforcing transparency in electoral processes and enhancing the right to information while emphasizing how informed electorate is crucial for the democratic process and is important in maintaining democratic integrity.

In addition to the above mentioned rulings, the Indian judiciary has played a crucial role in protecting women from various forms of violence and has consistently taken proactive measures to interpret and expand the scope of legislative provisions on the side of marginalized women. Though, in all societies, women and girls experience varying degrees of physical, sexual, and psychological abuse. Regrettably, India is notable for being one of the few countries where sexual violence has a detrimental impact on the lives of millions of women. Consequently, in order to tackle the multifaceted forms of violence against women, the Indian Govt. has implemented several strategies and enacted various legislations over time. In this regard, the contribution of female judges has been phenomenal in terms of their distinct viewpoint and sympathetic comprehension to instances involving discrimination and gender-based violence. For instance, in recent times, with female judges in the bench, substantial transitions have occurred from a restrictive understanding of the presumption of evidence concerning rape to a more robust dependence on the testimony provided by the victim regarding the occurrence of the rape. In some cases, the conviction has been established exclusively on the basis of victim testimony.

In the case of ‘*State (Govt. of NCT) of Delhi v Pankaj Chaudhary’* (2019), a panel of the Supreme Court, comprised of ‘J. Indira and J. R. Banumathi’, it has been determined that every human, regardless of their perceived moral character, possessed the entitlement to refuse participation in sexual intercourse. The ruling specifically addressed women’s rights and privacy. The consistent engagement in sexual activity by the victim does not offer substantial proof to show a deficiency in moral character. In the 2016 case of *‘Raja v. State of Karnataka’* (2016), a panel of judges consisting solely of males had deduced that the victim was ‘*accustomed to sexual intercourse’,* a finding that utterly disregards this inference. In a similar vein, in the case of ‘*Lakshmi Kanta Kamath v. The State of West Bengal*’ (2015), Justice ‘Indira Banerjee’ remarked that *‘a mere act of helpless resignation in the teeth of compulsion’* could not be considered consent in her dissenting opinion to Justice ‘Indrajit Chatterjee’s’ decision to acquit the rape accused in the absence of external injury marks on the victim’s body.

In another significant verdict of Supreme Court in State of ‘*Jharkhand v. Shailendra Kumar Rai*’ *(2022)*, Justice ‘Hima Kohli’, as a member of the Bench along with Justice ‘D.Y Chandrachud’, instructed the Union and state governments to take steps to eliminate any references to “*two Finger test*” from the medical curriculum. Furthermore, it declared that doctors found performing the test would be considered guilty of misconduct. Therefore, the court strongly protected the dignity and integrity of women, in accordance with the previous ruling of the Supreme Court in the case of ‘*Lillu @ Rajesh & Anr. V. State of Haryana’* (2013) wherein this test was held to be violative of the physical and mental well-being, as well as the dignity and privacy of the rape survivors.

In May 2011, the SC bench, including Justice ‘Gyan Sudha Mishra and Justice Markandeya Katju’, conclusively resolved the long-standing issue regarding euthanasia in the case of ‘*Aruna Ramachandra Shanbaug v. Union of India’* (2011). In this landmark ruling, the Bench, while deliberating upon euthanasia, affirmed the right to a dignified death. It also established the procedure for passive euthanasia, similar to other countries, and outlined the safeguards and guidelines to be followed in case of terminally ill patients, unable to give consent owing to being in a vegetative state or irreversible coma or being mentally incapacitated. The Court also suggested the practicality of removing section 309 of IPC that criminalized attempted suicide (*Aruna supra, para*, 100).

With an emphasis on the importance of dignity in life and death and fostering a greater awareness of the need for support rather than punishment for individuals suffering from severe distress, the repeal of Section 309 IPC by the newly enacted criminal law of the country, i.e., the Bhartiya Nyaya Samhita, 2023 is a major step in reforming India’s approach to mental health and suicide prevention following this case.

Thus, the above analysis of the judgments reveal that women’s participation in the judiciary has not only advanced gender equality on a broader scale apart from safeguarding the rights of women but has also contributed toward the establishment of a more equitable legal framework which aligns with principles of gender equality, social justice, sustainable development, and democratic values. Undoubtedly their significant contributions through their majority judgments as well through their dissents not only foster inclusivity, public trust and democratic integrity but also have been critical in pushing the boundaries of conventional social and judicial thinking, particularly in cases involving marriage, reproduction, divorce, gender equality, thus laying the foundation for a *New India* with better social and economic prospects for women and marginalized communities.

In addition to the aforementioned notable rulings, female judges have also played a significant role in leading crucial systemic and legislative transformations in India. Justice ‘Gita Mittal’, as the chairperson of the committee responsible for designing the ‘*Vulnerable Witness Project*’, ensured that witnesses would not be required to directly confront the accused. Instead, they could provide their testimony in a secure and private environment. This initiative ultimately resulted in the establishment of the first courtroom of its kind in Delhi in September 2012. Similarly, Justice ‘Leila Seth’, in her capacity of being a member of the ‘Law Commission of India’ [15th], played a crucial role in effecting revisions to the ‘Hindu Succession Act of 1956’. These reforms were aimed at safeguarding the inheritance rights of daughters in relation to ancestral property. In addition, as a member of the three-person Justice Verma committee established following the horrifying 2012 Delhi gang rape case, she played a crucial role in proposing expedited trials and stricter penalties for sexual offenses. Until 2004, Justice ‘Sujatha Manohar’ had a position as a member of the NHRC.

## 5 Vacuum in the appointment of gender-variant persons in the Indian judicial system

Due to numerous progressive judgments of the apex court advocating for inclusiveness and equal access to justice regardless of one’s socio-economic status and sexual orientation, there has been undoubtedly been a g*rowing acceptance of the LGBTQ community in urban India in the 21st century*. It is worth mentioning that it was the Supreme Court verdict in ‘*National Legal Services Authority v. Union of India’ (2014),* when trans genders for the first time were given recognition as a “Third Gender” and various directions were issued by the SC to the Central and State Governments to make laws with recognizing them as socially and educationally backward citizens and thereby extending them reservation in admission to educational institutions and appointments to public offices. In this case, the Court also directed the Government to frame necessary welfare policies for the community and to spread awareness so that they gain the necessary dignity and respect within the society, which is a fundamental right of every citizen of the country. In fact, after the NALSA judgment only, it became possible in India that one could self-identify as belonging to ‘third gender’ in Government-issued identity-cards such as Passports, Voter identity cards, Aadhar, PAN cards etc., as well as in university application forms and bank application forms. Later, according to the directives provided by the SC, the Indian government also passed the ‘Transgender Persons (Protection of Rights) Act, 2019’ and provided them the right to self-perceived gender identity [‘Transgender Act’, section 4 (2)]. Similarly, in the case of ‘*Navtej Singh* (2018), the Apex Court, while decriminalizing consensual same-sex relationships, earmarked the need for enacting more laws to sensitize society and create an environment that was conducive to the growth of the gay community.

In fact, apart from the contribution of constitutional courts in securing their progress, indeed it is worth noting their statutory recognition in terms of their identity and sexual orientation under the newly enacted inclusive criminal Laws of the country, wherein they have not only been recognized as a victim of various crimes committed under the ‘Bhartiya Nayaya Sanhita’ (BNS) but their sexual orientation too has been acknowledged with decriminalization of consensual same-sex relationships. However, despite all these measures and developments, their battle toward recognition of their rights, civil liberties and their social upliftment is long drawn and can still be witnessed. The unanimous decision of the five-judge bench of the Supreme Court in the case of *Supriyo Chakraborty’* (2023) headed by Chief Justice of India (CJI) ‘DY Chandrachud’, against legalizing same-sex marriage in India accepting the arguments put forward in opposition by the Govt. of India not only represents a clear setback for the LGBTQ+ community on account of unwillingness of both judiciary and legislature in securing them their basic human right, but the existence of this transphobic gender binary outlook also calls for an intensive inquiry into the factors having the potential to impact their social reintegration, extending the same to the fact whether their inclusiveness recognized over the years encompasses their equal representation in the judiciary as well or not. A probe into this reveals a sad reality that the Indian judiciary is no exception, with only three LGBTQ+ community Judges at present and no transgender judge in the Constitutional Courts of the Country. The appointment of India’s first *transgender Judge* Joyita Mondal in West Bengal (Aishik, 2017), followed by the appointment of Vidya Kamble in Nagpur (Sahitya, 2018) and Swati Bidhan Baruah in Assam undoubtedly marks a significant victory for the transgender community, proving that trans people are worthy of every right as any other human being from a binary gender system (Sengar, 2018). However, with their minuscule representation in judicial institutions on one hand and their complete absence in forming bench in Constitutional Courts, underlines the need for reservations in Government jobs for the members of this community as a step toward their inclusiveness into the mainstream society. At this juncture it becomes pertinent to draw attention toward the reluctance on the part of the government in India to appoint an openly gay man Mr. ‘Saurabh Kripal’ as a judge of the High Court of Delhi (Times of India, 2023). Despite the name being approved by the SC collegium, the Government seems to be totally oblivious to the principle of inclusiveness, the ambit of which needs expansion, reflecting diversity in rendering justice with sensitivity to the needs of the Gender Variant community also.

## 6 Conclusion and suggestions

Therefore, the above made discussion clearly demonstrates as to how Indian Courts, led by female judges, have assumed the role of a catalyst for societal reforms resulting in transformative conversations, such as subjecting gender-biased laws, policies, and norms to Constitutional scrutiny and invalidating laws that harm women in addition to devising innovative solutions to address systemic injustice and the exploitation of women. The presence of women judges in the Indian judiciary has not only paved the way for creating a just and gender-equal society, but has also shown to influence the development and interpretation of laws to be more inclusive and reflective of women’s experiences. Hence, endeavors should be made to enhance their presence in the Indian judiciary which represents the population’s diversity and is more likely to be perceived as fair and just and which could be commenced with implementation of horizontal reservation for women in the superior judiciary, including subordinate courts and at the same ensuring that merit remains uncompromised.

Similarly, in the wake of the recognition of alternative sexualities, it also becomes imperative to understand that the three pillars of Government need to function in harmony and it is only then that the shared goal of an inclusive and an equitable society would be achieved. As mentioned supra, various legislative and judicial reforms indeed set the tone for inclusiveness of LGBT+ community and are worth celebrating. However, despite all these measures, the LGBT+ community is battling hard to get recognition of their legitimate rights, civil liberties and their quest for social upliftment. The denial to conferring legitimacy to same-sex marriage and the reluctance toward appointment of persons of this community as judges at higher levels has contributed to their miseries. Persons belonging to this community need to go that extra mile in order to secure the support which they deserve in a society wherein their inclusion in the process of imparting justice might just bring a shift in the progressive thinking which is much needed to accord true sense of equality to these persons. This recognition will prove crucial in recognizing their talent, their abilities and their potential to contribute to the judicial system by rendering influential judgments which may more accurately reflect the diverse range of experiences within India. The establishment of a more gender-diverse bench will not only lead to a more inclusive judicial system but will also help in ensuring a robust legal system that aims at promoting true gender justice and equality at all levels of society.

Further, continued efforts through various training programs and workshops, along with gender sensitization of judicial officers toward gender issues coupled with the much required infrastructural changes in Courts to make them more gender inclusive, are also necessary in order to achieve this vision.

## Data Availability

The original contributions presented in the study are included in the article/supplementary material, further inquiries can be directed to the corresponding author.

## Appendix: Case laws

- *ADM Jabalpur v. Shivkant Shukla* (1976) SCC (2) 521
- *Ak Gopalam v. State of Madras* (1950) SCC 228
- *Amish Devgan v. Union of India* (2021) 1 SCC 1
- *Aruna Ramachandra Shanbaug v. Union of India* (2011) 4 *SCC* 454
- *Independent Thought v. Union of India (AIR 2017 SC 4904)*
- *Indian Young Lawyers’ Association v. The State of Kerala* (2019) 11 SCC 1
- *Jayachandera v. Aneel Kaure* (2005) 2 SCC 22
- *Jharkhand v. Shailendra Kumar Rai* (*2022 SCC Online SC 1494*)
- *Joseph Shine v. Union of India* (2018) 11 SCALE 556
- *Kaushal Kishor v. State of Uttar Pradesh & Ors* (2023) LiveLaw (SC) 4
- *Kharak Singh v. State of UP* (1964) 1 SCR 332
- *Lakshmi Kanta Kamath v. The State of West Bengal* (2015) Lawsuit (Cal) 129
- *Laxmi v. Union of India* (2015) 2014 SCC 2427
- *Lillu @ Rajesh & Anr. V. State of Haryana* (2013) 14 SCC 643
- *Mankayi v AngloGold Ashanti Ltd (CCT 40/10) [2011] ZACC 3*
- *Minister of Home Affairs v Fourie (2005 ZACC 19)*
- *Mrs. X v. GNCTD & Anr* 2022/DHC/005344
- *Mukesh & Anr v. NCT of Delhi & Ors*. (2017) 6 SCC 1
- *National legal Services Authority v. Union of India* (2014) 5 SCC 438
- *Navtej Singh v. Union of India* (2018) *10 SCC* 1
- *Neetu Singh v. Telegram* (2022) SCC Online Del 2637
- *Obergefell v. Hodges | 576 U.S. 644 (2015)*
- *People’s Union for Civil Liberties v. Union of India* (2013) 10 SCC 1
- *Pratibha Gaur v. NCT of Delhi* (2021) 12 DEL CK 0228
- *Pyla Mutyalamma v. Pyla Suri Demudu c*ase@ *Satyavathi* 2011 (9) SCALE 403
- *Raja v. State of Karnataka* 2016 (10) SCC 506
- *Rattan Chand v. Askar Nawaz Jung Hira Chand* 1991 SCR (1) 327
- *Sarla Mudgal v. Union of India (AIR 1995 SC 1531)*
- *Smt. Chetna Rathee v. Sh. Chahit Kundu* CM(M) 34/2023 & CM APPL. 1100/2023
- *Smt. Pratibha Singh v. Mr. Vineet Kumar* W.P.(C) [6986/2023 & CM APPLs. 27185–86/2023]
- *State (Govt. of NCT) of Delhi v Pankaj Chaudhary* (2019) 11 SCC 575
- *State of Madhya Pradesh v. Jogendra & Anr* 2022 SCC Online SC 33
- *Suchita Srivastava v. Chandigarh Administration [2009] 13 SCR 989*
- *Supriyo Chakraborty v. Union of India* 2023INSC 920.
- *Vinitha Saxena v. Pankaj Pandit* (2006) 3 SCC 78
- *Vishaka and Ors. v. State of Rajasthan and Ors* AIR 1997 SC 3011
- *Vivek Narayan Sharma v. Union of India* (2023) SCC OnLine SC 1
- *Vriend v Alberta [1998] 1 SCR 493*
- *Whole Woman’s Health v. Hellerstedt 579 U.S. 582 (2016)*
